# Characterising Online News Comments: A Multi-Dimensional Cruise Through Online Registers

**DOI:** 10.3389/frai.2021.643770

**Published:** 2021-06-14

**Authors:** Katharina Ehret, Maite Taboada

**Affiliations:** Discourse Processing Lab, Department of Linguistics, Simon Fraser University, Burnaby, BC, Canada

**Keywords:** register variation, online news comments, online language, multi-dimensional analysis, corpus linguistics

## Abstract

News organisations often allow public comments at the bottom of their news stories. These comments constitute a fruitful source of data to investigate linguistic variation online; their characteristics, however, are rather understudied. This paper thus contributes to the description of online news comments and online language in English. In this spirit, we apply multi-dimensional analysis to a large dataset of online news comments and compare them to a corpus of online registers, thus placing online comments in the space of register variation online. We find that online news comments are involved-evaluative and informational at the same time, but mostly argumentative in nature, with such argumentation taking an informal shape. Our analyses lead us to conclude that online registers are a different mode of communication, neither spoken nor written, with individual variation across different types of online registers.

## 1 Introduction

We present a text-linguistic study of the characteristics of online news comments as compared to other online registers. In contrast to many other registers on the web, online news comments have so far not been thoroughly scrutinised. However, there has been a sense, among journalists (Woollaston, 2013; McGuire, 2015) and researchers alike (Godes and Mayzlin, 2004; Marcoccia, 2004; North, 2007), that online news comments are like conversation or dialogue. We have challenged this assumption, in a related article comparing online news comments to face-to-face conversation and other traditional registers: While online news comments were found to contain features of personal involvement typical of face-to-face conversation, they can best be described as a type of written, evaluative discourse (Ehret and Taboada, 2020). As a matter of fact, we argue that online news comments should be regarded as their own register, and that language on the web, in general, is quite different from either standard written or spoken language (Ehret and Taboada, 2020, 23–24). It is natural to describe new registers in terms of other, more familiar registers, which is perhaps what leads to the characterisation of online news comments as conversations. This label has also sometimes been applied to blogs, but has also been found inadequate, as Peterson (2011) has argued. In his analysis of blogs, Peterson found that, although blogs have an expressive potential, such potential is not realised in the same way as it is in conversation.

An ever-growing body of research analyses online language in general (e.g., Crystal, 2011; McCulloch, 2020), specific online registers, such as email (Frehner, 2008; McVeigh, 2020), blogs (Herring et al., 2004; Peterson, 2011), reviews (Taboada, 2011; Vásquez, 2014), Facebook (West, 2013; Farina, 2018), Twitter (Zappavigna, 2012; Clarke and Grieve, 2019), or online and social media language in general (Giltrow and Stein, 2009; Titak and Roberson, 2013; Page et al., 2014; Biber and Egbert, 2016; Berber Sardinha, 2018; Biber and Egbert, 2018). Little attention, however, has been paid to the linguistic characteristics of online news comments, a register now ubiquitous in our interactions with news, whether on the pages of newspapers or through platforms such as Twitter and Facebook.

Against this backdrop, the present paper explores the structural linguistic properties of online news comments in comparison with other online registers such as travel and opinion blogs, interactive discussions and news reports, or advice pieces, since our previous analysis involved a traditional written and spoken corpus. We will thus establish what—if not like spontaneous conversation—online news comments are like in the context of other online registers. The data for our analysis is drawn from the comments section of the *Simon Fraser University Opinion and Comments Corpus* (SOCC) on the one hand, and the *Corpus of Online Registers of English* (CORE) on the other. SOCC is the largest corpus of online comments publicly available, while CORE is to date the largest available corpus of registers on the web. Methodologically, we conduct a multi-dimensional analysis (Biber, 1988), considering a comprehensive set of well-established lexico-grammatical features, to describe online news comments along the emerging dimensions of variation in our dataset.

Our analysis shows that multi-dimensional analysis (MDA) is very well suited to capturing the variation found in some common online registers. By applying the part-of-speech tag frequency statistics and dimensionality reduction characteristic of MDA, we are able to place online news comments in a unique space as compared to other online registers. To be more precise, we find that there are three dimensions along which online news comments can be described in online variational space, with two of them being most prominent. The first dimension, which we labelled “Involved-evaluative” points to the involved nature (in the Biberian sense; Section 3) of online registers and online comments, with an involvement that includes evaluative meaning. We find, however, that the most characteristic dimension is “Informational-argumentative”, marked by information density (nominalisations, longer words) and argumentative features such as conjuncts. Finally, the third, minor dimension, “Narrative-descriptive vs. instructional” supports our analyses of the first two, showing an involved personal narrative mixed with instructional detail.

The paper is structured as follows: Section 2 describes the data source and methodology. In Section 3 the results of the MDA analysis are presented. Section 4 discusses online news comments in light of the results. Section 5 offers a brief summary and concluding remarks.

## 2 Material and Methods

### 2.1 Online News Comments and Other Online Registers

Our aim is to compare online comments to other, well-studied online registers. To that end, we use the *Corpus of Online Registers of English* (CORE), the largest, most diverse corpus of online language currently available (Biber et al., 2015; Egbert et al., 2015; Biber and Egbert, 2018). CORE was conceived as an attempt to classify various online registers. The data was obtained by sampling publicly-available documents and tagging them in a bottom-up process. About 50,000 web documents were labelled through crowd sourcing, resulting in six general (written) register types and several sub-registers. The general registers were provided by the researchers, but the sub-registers were crowd sourced and labelled by users according to guidelines (Biber et al., 2015). Registers were labelled according to their communicative purpose: to narrate events, describe or explain information, express opinion, persuade, explain instructions, or to express oneself through lyrics. Many of the sub-registers were deemed to be hybrid, because they include characteristics of more than one register or sub-register. CORE thus comprises, for instance, sub-registers (with main register in parentheses) such as personal blog (narrative), FAQ (description), review (opinion), description for sale^1^ (persuasive), recipe (instructional), or song lyrics (lyrical).

We chose CORE because of its focus on the public web, the readily available set of registers that one is likely to encounter online. An additional set of computer-mediated communication exists, including text messages (SMS, WhatsApp, Telegram, Signal, Direct Messages on Facebook or Twitter, etc.,), but that tends to be a one-to-one or small-group type of communication, not one to be publicly displayed the way online news comments are.

From this varied source of online materials, we select a large sample, excluding registers that are not unambiguously defined or not directly comparable to the online news comments we are interested in. In this vein, we exclude all hybrid registers, registers labelled as “other”, lyrical and fully narrative registers, i.e. short story, historical article, and narrative, as well as spoken material. The sample does include typical online registers such as personal blog, travel blog, or news report, which are also labelled as narrative in CORE. In general, the sampling criterion excluded registers that may appear outside of the internet (short stories), but included online-only registers (travel blog), even when they were both under the same macro-register (narrative). This sample of CORE amounts to 43.7 million words (Table 1).

**TABLE 1:** Overview of analysed registers, corpus source, and number of words.

Register	Sub-register	Corpus	Word count
Narrative	Personal blog	CORE	3,264,463
—	Travel blog	CORE	382,124
—	Sports report	CORE	2,729,925
—	News report/blog	CORE	9,806,239
Informational description	FAQ	CORE	678,562
—	Description of a person	CORE	958,925
—	Informational blog	CORE	2,141,271
—	Encyclopedia article	CORE	1,613,338
—	Research article	CORE	1,905,846
Opinion	Opinion blog	CORE	10,898,872
—	Advice	CORE	1,415,912
—	Religious sermon/blog	CORE	1,435,058
—	Review	CORE	2,121,213
Persuasive	Description for sale	CORE	1,130,813
Instructional	Recipe	CORE	89,513
Interactive discussion	Interactive discussion	CORE	3,099,725
Online news comments	—	SOCC	5,779,157
**Total**	—	—	**49,450,956**

The online news comments come from the comments section of the *SFU Opinion and Comments Corpus* (SOCC), a large dataset of comments posted on the website of the Canadian English-language newspaper *The Globe and Mail* (Kolhatkar et al., 2020). The corpus contains more than 660,000 comments, a rough total of 37 million words. In this paper we specifically analyse comment threads, sequentially posted comments with a seemingly conversation-like structure, rather than individual comments. The analysis is furthermore restricted to comment threads with a minimum of 700 words, to improve the robustness of the multi-dimensional analysis (cf. Ehret and Taboada, 2020, 6). The comment threads were then analysed as individual comments, for a total of 5,949 comments. This selection of the SOCC corpus contains 5.8 million words and 388,141 sentences (but note that sentence boundaries are imprecise due to the online and informal nature of the data).

We should point out that we analyse comment threads rather than individual comments. This is in part due to technical considerations, because multi-dimensional analysis requires texts of a certain length, with 400 words the most common minimum length in the literature (Biber, 1995). There are also methodological considerations, in that what we are studying is the nature of online comments, which are typically posted in sequential form and constitute a thread of ideas and contributions. The drawback of this method is that the communicative function of one comment may be different from the next comment. We treat the entire thread as a communicative event, just like spoken conversations which include more than one participant.

### 2.2 Multi-Dimensional Analysis

Multi-dimensional analysis (MDA), originally introduced by Biber (1988) to describe variation in written and spoken registers of English, is a multi-variate statistical technique and the classic tool in text-linguistic approaches to register variation. MDA employs exploratory factor analysis to determine the shared variation in a given dataset based on the co-occurrence frequencies of linguistic features. The extracted factors are then interpreted as dimensions of variation according to the functional-communicative properties of the most important linguistic features on each factor.

We conduct a multi-dimensional analysis of our dataset largely following the statistical recommendations outlined in Biber (1988, 71–93), which we have also employed and detailed in previous work (Ehret and Taboada, 2020, 7–11). This paper differs from our previous work in that it focuses specifically on online language. To be more precise about the methodology, we apply maximum likelihood factor analysis as available in the R *stats* package and utilise a promax factor rotation. All statistics, unless otherwise indicated, were performed in R (R Core Team, 2020). The scripts, all statistics described here and elsewhere in the paper, additional statistical material, and data are available on GitHub.^2^

The linguistic features analysed in this paper consist of 67 core grammatical features of English customarily utilised in MDA studies (Biber, 1988; Biber and Finegan, 1989; Pavalanathan et al., 2017; Clarke and Grieve, 2019). These features include, but are not limited to, modals, pronouns, subordination and coordination, tense and aspect markers, as well as some special verb classes (Biber, 1988, 221–245). The dataset was automatically annotated with part-of-speech tags for these features using the *Multi-dimensional Analysis Tagger*, version 1.3.2 (Nini, 2019), a replication of Biber’s (1988) original MDA tagger.^3^ The part-of-speech tags and corresponding features are listed in Supplementary Table S1 in the supplementary material. Subsequently, the occurrence frequencies of the 67 features were automatically retrieved, and normalised per 1,000 word tokens using a custom-made python script (available from our GitHub repository; see Data Availability Statement at the end of the paper). The features type-token-ratio (TTR) and average word length (AWL) were not normalised. Type-token ratio was calculated for the first 400 words in each text file, and average word length was calculated by dividing the number of orthographic characters by the number of tokens in each text file.

**FIGURE 1:**
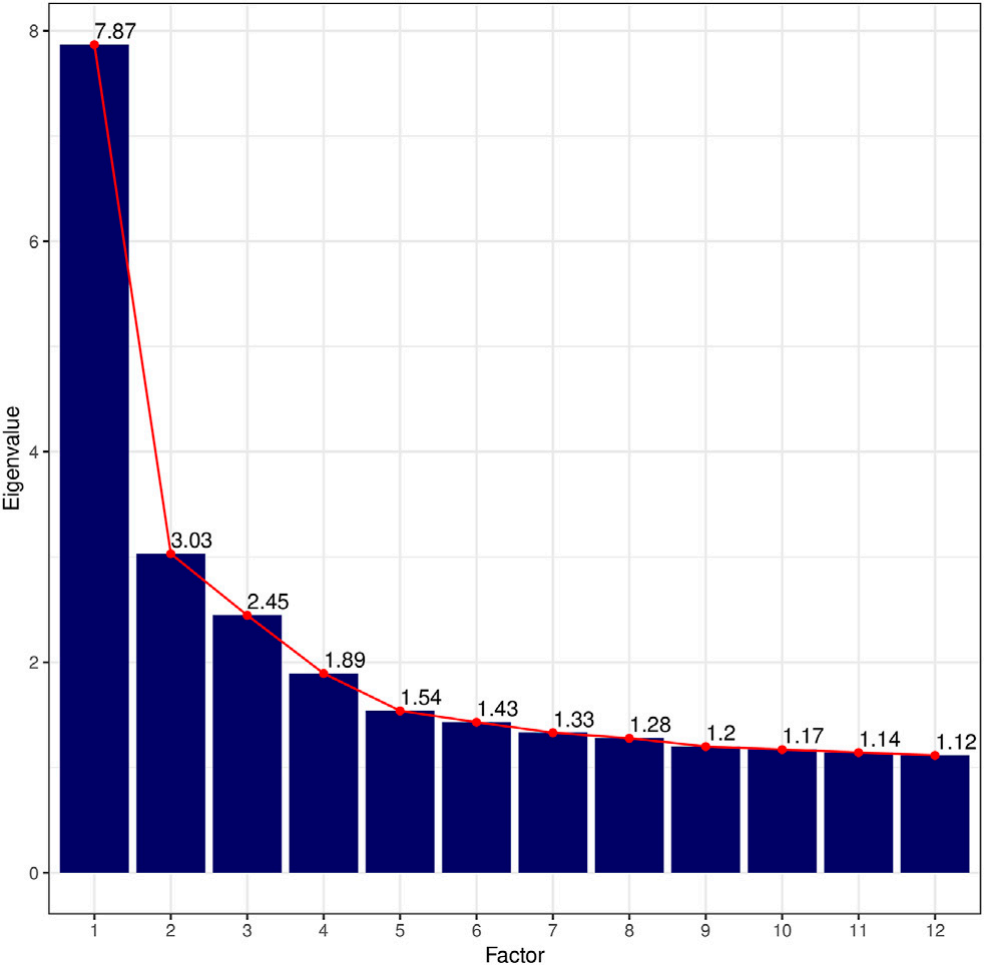
Screeplot of eigenvalues for the first twelve factors. Eigenvalues were rounded to the second digit.

With an overall measure for sample adequacy of 0.77 and a *p*-value = 0 for *Bartlett’s Test of Sphericity,* our dataset is statistically suitable for conducting a factor analysis (Dziuban and Shirkey, 1974, 358–359). After inspecting the screeplot of eigenvalues in Figure 1, which shows a first break after the third factor before flattening out into a straight line, and the linguistic interpretability of the factors, we extract three factors for the final model (Supplementary Table S2). Traditionally, a factor is regarded as linguistically interpretable if it comprises at least five salient loadings. Following Biber (1988, 87), we consider loadings with a conservative cut-off ≥ |0.3| as statistically significant and hence salient. Note that Factor 3 is not fully linguistically interpretable according to these criteria, because it only comprises four salient loadings. However, it is included in the final model in order to avoid conflating factors, and to enhance the interpretability of the other factors in the model. Furthermore, for a tentative interpretation of Factor 3, we consider secondary features with loadings ≥ |0.2|. The total variance explained by the final model is about 20%.^4^

Finally, factor scores are automatically calculated for each text in the dataset. Factor scores indicate the position of each text on a given factor: the higher the absolute value of a factor score for a given text on a specific factor, the more typical is this text for the factor and the underlying linguistic dimension represented by the factor (Biber, 1988, 93). Additionally, factor scores also indicate on which pole of a factor a given text is to be positioned. Positive factor scores indicate that a given text weighs on the positive pole of a specific factor while negative scores indicate that a given text weighs on the negative pole of a specific factor. Consider, for instance, the text with the filename 19_N_personal_1747770_MAT.txt which belongs to the register personal blog. This text has a factor score of 2.36 on the first factor and a factor score of −0.85 on the second factor. On the basis of these factor scores, we can conclude that this text is more typical of Factor 1 than of Factor 2. Furthermore, the text contains many of the linguistic features which load high on the positive pole of Factor 1 and is marked by the absence of linguistic features which load high on Factor 2 (a detailed interpretation of the factors is given in Section 3).

In addition to factor scores, we calculate scores to position the individual registers as a whole on each factor. These scores are referred to as “mean factor scores” in this paper and are calculated as the arithmetic mean of the factor scores for all texts pertaining to a given register (Table 2).

**TABLE 2:** Mean factor scores per register. Positive values indicate that a register weighs on the positive pole of a factor; negative values indicate that a register weighs on the negative pole of a factor. All values were rounded to four decimal points.

Register	Factor 1	Factor 2	Factor 3	Factor 4
Advice	0.6092	−0.9682	0.3232	0.1130
Comments	0.1365	−0.0955	0.4659	0.4057
Description of a person	−0.5195	0.8685	−0.4012	−0.7035
Interactive discussion	1.1239	−0.2665	−0.3428	−0.0081
Encyclopedia article	−0.9321	0.2353	0.1470	−0.5167
FAQ	−0.4068	−1.1419	0.6247	0.1849
Informational blog	−0.4021	−0.6828	0.3098	0.2375
Description for sale	−0.4454	−0.8685	−0.3528	0.3339
News report	−0.5372	0.3953	−0.0345	−0.1031
Opinion blog	0.0520	−0.1194	0.2703	0.0676
Personal blog	0.9369	0.2646	−0.3415	−0.5101
Recipe	0.5258	-0.8007	−1.1673	−0.2321
Religious sermon	0.2640	0.1920	0.1669	−0.1248
Research article	−1.7230	−0.0744	1.2602	−0.1149
Review	0.1603	−0.3214	−0.4674	0.3110
Sports report	0.2669	0.3684	−0.8110	−0.2196
Travel blog	0.2887	0.0542	−0.6574	−0.5771

## 3 Dimensions of Linguistic Variation Online

In this section, the extracted factors are interpreted as dimensions of variation. This means that each factor is linguistically interpreted based on the co-occurrence and complementary distribution of linguistic features and their shared functional-communicative properties (Biber, 1988, 91–92). Specifically, features with loading |≥ 0.3| are given the greatest importance in this interpretation, yet secondary features with less salient loadings are also considered. Features which load on multiple factors with the same polarity are primarily considered on the factor where they load highest. This interpretation is aided and confirmed by analysing the distribution of registers across the various dimensions. Table 3 provides a summary of the three factors (for a complete list of features and loadings, see the GitHub repository in footnote 2).

**TABLE 3:** Overview of the three factors including features with loadings ≥ |0.3|. Positive loadings indicate co-occurrence of the features; negative loadings indicate complementary distribution.

**Factor 1**		**Factor 2**	
**Involved-evaluative**		**Informational-argumentative**	
Contractions	0.735	Nominalisations	0.716
First person pronouns	0.708	Average word length	0.652
Adverbs	0.599	THAT verb complement	0.355
Analytic negation	0.571	Conjuncts	0.347
Present tense	0.555	Attributive adjectives	0.319
BE as main verb	0.547	—	—
Pronoun IT	0.484	No negative features	—
Private verbs	0.46		
Emphatics	0.449		
Second person pronouns	0.445	**Factor 3**	
Conditional subordinator	0.423	**Narrative-descriptive vs. instructional**	
DO as proverb	0.398	Past tense	0.983
Predicative adjectives	0.35	Third person pronouns	0.375
THAT deletion	0.334	Public verbs	0.321
Demonstrative pronouns	0.33	—	—
—	—	Present tense	−0.523
Average word length	−1.036		
Nouns	−0.737		
Nominalisations	−0.706		
Prepositions	−0.64		
Attributive adjectives	−0.497		
Phrasal coordination	−0.462		
Past participle WHIZ deletion	−0.379		

The factors in our analysis and the emerging dimensions for this particular set of online registers vary from those that have been proposed for the CORE corpus by Biber and Egbert (2018). In their analysis, Biber and Egbert explore the entire CORE corpus, which, as we mention in Section 2.1, includes hybrid registers and spoken registers. Their first dimension, for instance, is thus “Oral-involved vs. literate”, which captures the differences between song lyrics, TV dialogue, and interactive discussions on the one hand, and written registers such as research articles and encyclopedia entries on the other. Our dataset is a different one and, consequently, the emerging dimensions capture variation of online registers that are closer in nature to online news comments.

Factor 1 comprises 15 positive and seven negative features with salient loadings ≥ |0.3| and is therefore the most clearly defined factor. On the positive pole of the factor, we find features which are typical of spontaneous, informal, and involved communication such as contractions, first and second person pronouns, analytic negation, the pronoun *it*, private verbs which express personal attitudes or emotions (e.g. *believe*, *decide*, *know*), and emphatics (Biber, 1988, 105–106). In addition, some of the most salient features are not only well known as characteristic of spontaneous spoken language (Biber, 1988, 228–229), but have also been recently identified as markers of evaluation and opinion in online news comments (Ehret and Taboada, 2020, 13): *be* as main verb, adverbs, and predicative adjectives. Together, these three features often occur in constructions which are typically used to convey evaluation (White, 2003; Hunston, 2011), such as in Example (1).

(1)  a. It’s_be main verb_ not ideal_predicative adjective_ for my husband                  […] (personal blog, 19_N_personal _0000263_MAT.txt).

     b. This is_be main verb_ sometimes_adverb_ hard_predicative adjective_ to                  conjure up when you have been woken numerous times in                  the night to feed. (advice, 10_O_advice_3360949_MAT.txt).

On the negative pole of Factor 1, we find features which are well known as characteristic of an informational and abstract style in English: average word length, nouns, nominalisations, attributive adjectives, and prepositions are all indicators of information density and lexical specificity and are common in scientific or academic writing (Biber, 1988, 104–105). All in all, Factor 1 strongly resembles the Dimension “Involved vs. informational production” identified in Biber (1988) with, one could argue, an evaluative slant. We therefore interpret Factor 1 as Dimension 1 “Involved-evaluative vs. informational” and we shorten it to “Involved-evaluative” in the rest of the paper. In work by Biber and colleagues, multiple registers across different languages have been shown to be distributed across two main axes, involved vs. informational. The involved dimension refers to language use that includes “affective, interactional, and generalized content”, as opposed to language with “high informational density and exact informational content” (Biber, 1988, p. 107).

**FIGURE 2:**
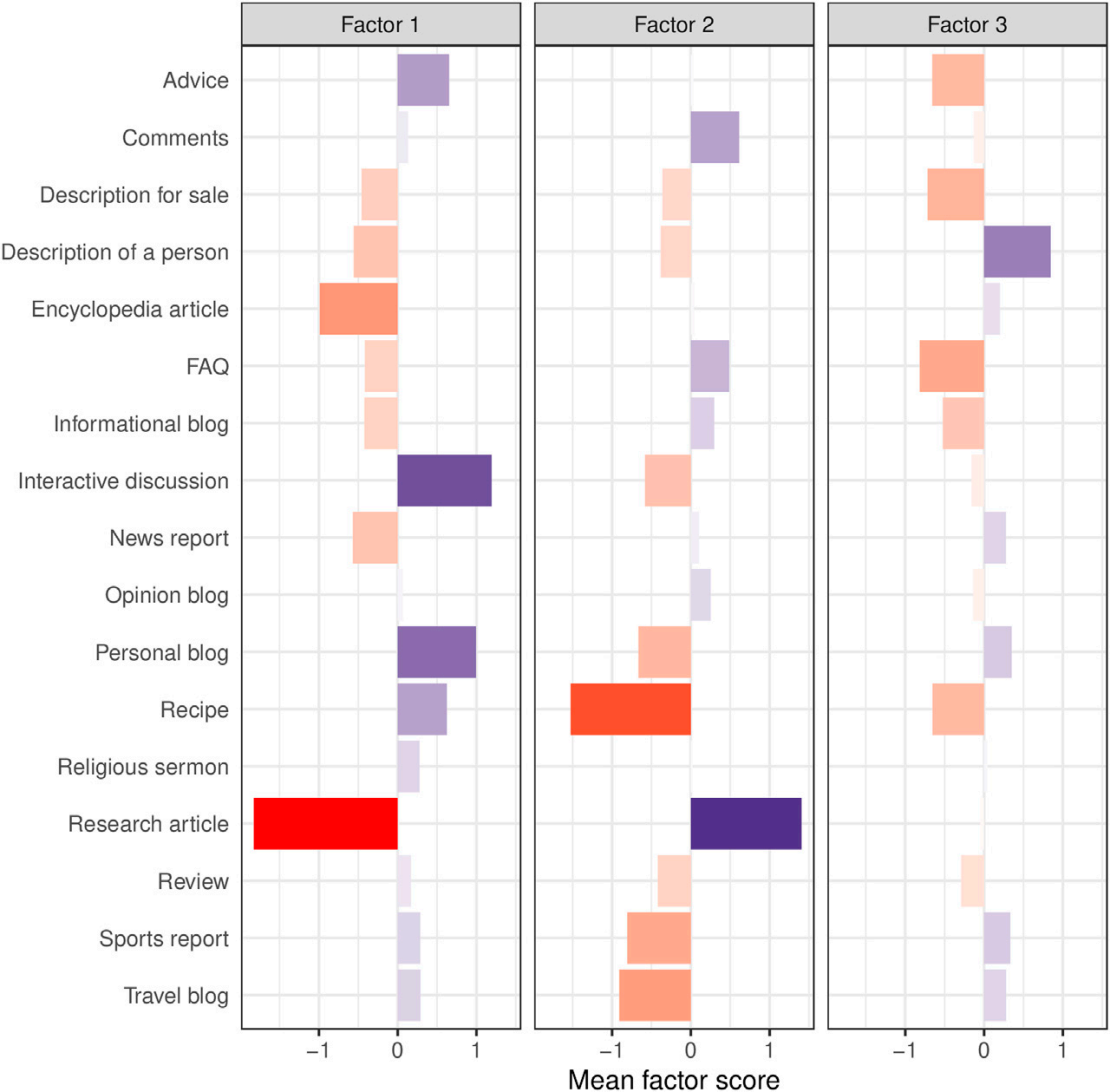
Register distribution across the three factors/dimensions. Colour intensity indicates strength of mean factor scores. Red bars indicate negative values; blue bars indicate positive values.

This interpretation for Factor 1 dovetails with the distribution of registers on Dimension 1 (Figure 2). For instance, research and encyclopedia articles are located on the negative pole, while personal blogs and interactive discussions are representative of the positive pole of Dimension 1. Note that, in contrast to Biber’s original Dimension 1, the dimension presented in this paper does not represent the fundamental distinction between written and spoken language. Instead, all registers analysed in this paper are written, and Dimension 1 thus distinguishes between online written discourse which is involved and evaluative and online written discourse which is informational (and presumably constructed as objective).

In contrast to the first factor, Factor 2 is defined exclusively by positive features. The five salient positive features are nominalisations, average word length, *that* verb complement, conjuncts, and attributive adjectives. The co-occurrence of nominalisations, high average word length, conjuncts, and attributive adjectives are indicators of information density and information integration. Nominalisations can also be interpreted as conveying specialised or abstract information (Biber, 1988, 227) such as, for instance, in scientific discourse. Conjuncts (e.g. *however*, *on the other hand*) are also prominent markers of argumentation and coherence (Halliday and Hasan, 1976; van Eemeren et al., 2007; Tseronis, 2011; Kolhatkar and Taboada, 2017b) as exemplified in (2-a). The argumentative aspect of Factor 2 is stressed by the secondary non-salient feature suasive verbs (feature loading 0.296) which express varying degrees of persuasion such as *propose*, *suggest*, or *allow*, but also future intent and certain speech acts (e.g. *ask*) (see Quirk et al. (1985) for a full list). In combination with *that* verb complements, we interpret them as markers of argumentative discourse with the aim to promote ideas, make an argument, or persuade an audience, as in Example (2-b). A look at the distribution of registers confirms this interpretation. Research articles are the most representative register on this factor, followed by FAQ and comments. Factor 2 is thus dubbed Dimension 2 “Informational-argumentative”.

(2) a. These are issues of jurisdiction, however_conjunct_, not privacy. (comments, comments_28791923 _54_MAT).

b. He proposes_suasive verb_ that_that verb complement_ an individual might be genetically predetermined […] (research article, 31_IDE_res_0026415_MAT.txt).

Factor 3 counts only four features with loadings ≥ |0.3|, and can, strictly speaking, not be fully and reliably linguistically interpreted. The interpretation provided here is therefore a tentative one, but we believe it is useful, as it supports the interpretation of the first two factors. Past tense, third person pronouns, and public verbs load on the positive pole of Factor 3 and are clear indicators for a narrative style (Biber, 1988, 108). Furthermore, the non-salient feature *that* deletion with a loading of 0.264 suggests description or elaboration of information—although this feature is common in spontaneous production (Biber, 1988, 244). Representative registers on the positive pole of Factor 3 are description of a person, personal blog, and sports report. Such registers describe or narrate events, actions, or people in a spontaneous or informal fashion and thus correspond to the co-occurrence of the positive features described above.

There is only one salient negative feature on Factor 3: present tense. According to the literature, present tense usually occurs in spontaneous and involved discourse. To derive at a more dependable interpretation, we examine secondary, non-salient features with loadings | ≥ 2| which do not load higher with the same polarity on another factor. These features consist of second person pronouns (−0.26) and modals expressing possibility (−0.206). Together with present tense verbs, they can serve to convey instruction, direction, or advice as illustrated in Example 3. As a matter of fact, the most characteristic registers on the negative pole of Factor 3 are FAQ, description for sale, advice, and recipe. Factor 3 is thus tentatively labelled as Dimension 3 “Narrative-descriptive vs. instructional”.

(3) a. If the feta is_present tense_ more salty than sharp, you_2nd person pronoun_ may_possibility modal_ want to squeeze over a little lemon juice (recipe, 07_I_recipe_1478719_MAT.txt). 

b. If you_2nd person pronoun_ ’re_present tense_ expecting some kind of fairy tale ending, you_2nd person pronoun_ can_possibility modal_ forget_present tense_ about that right now. (description for sale, 16_IP_sale_0010352 _MAT.txt).

All together, these three factors paint a clear picture of the nature of online comments and online registers. We find an involved vs. informational divide, a result that has consistently been found in multi-dimensional analyses to be a feature of most registers, including cross-linguistically (Biber, 1995), and thus proposed as a universal of register variation (Biber, 2014). In our case, that first dimension is also imbued with evaluative meaning, conveyed by *be* as a main verb and predicative adjectives, which is why we have characterised that Factor as “Involved-evaluative”.

The “Informal argumentation” label for Factor 2 will be familiar to anyone who has spent any time online. One is likely to encounter vast amounts of argumentation, often involving a passionate defence of somebody’s choice of movie, book, video game, or other artistic productions and consumer products. Argumentation, of course, is often deployed to defend or attack political ideas, argue for and against the conspiracy theory *du jour*, or to praise and vilify public figures. The web is an opinionated space and comments on news even more so. This is what Tufekci (2008) has described as the expressive internet.

Finally, Factor 3 points to the helpful and friendly aspects of the internet, a place where we can encounter descriptions and personal narratives, together with extremely helpful advice on the most esoteric or mundane aspects of everyday life, the instrumental internet (Tufekci, 2008). We can personally attest to the usefulness of the internet’s collective wisdom when it comes to answering programming questions, solving plumbing issues, or fixing a bike. This factor combines that friendly aspect together with the construction of certain social personas (the helpful advice-giver, for instance).

## 4 Online News Comments Compared to Other Online Registers

**FIGURE 3:**
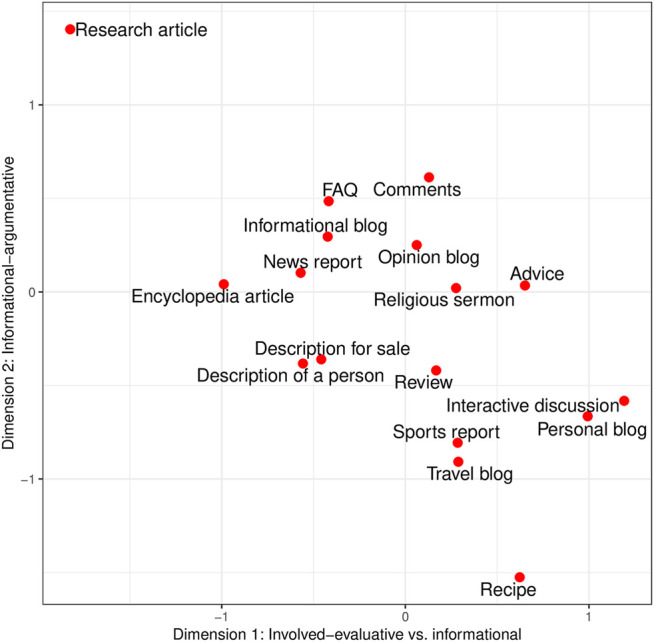
Two-dimensional view of register distribution along the most prominent dimensions. Note that negative values on Dimension 1 represent the informational pole while positive values on Dimension 1 represent the involved-evaluative pole.

After having interpreted the various factors as dimensions of variation, we will now turn to discussing the position of online news comments on the three dimensions relative to other online registers. Figure 3 provides a two-dimensional view of the analysed registers along the major dimensions: Dimension 1 “Involved-evaluative” on the x-axis and Dimension 2 “Informational-argumentative” on the y-axis. For strength and direction of mean factor scores and register distribution, see also Figure 2.

On Dimension 1, online news comments (mean factor score 0.129) are positioned on the positive pole, i.e., they are mainly characterised by the joint occurrence of involved and to some extent evaluative features such as contractions, first and second person pronouns, adverbs, predicative adjectives, and *be* as main verb. However, in comparison to other online registers, online news comments exhibit comparatively few of these features. Registers such as interactive discussion, personal blog, advice, and recipe, for instance, are much more involved in nature than online comments. Thus, while online comments are positioned on the positive pole of Dimension 1, they also contain a fair amount of informational-abstract features such as average word length, nouns and nominalisations, prepositions and attributive adjectives—this can also be seen from their location on Dimension 2 (see below). The registers most closely positioned or similar to online news comments on the positive pole of Dimension 1 are review (mean factor score 0.168) and opinion blog (mean factor score 0.062). On the negative pole of Dimension 1, the most similar registers to the comments are FAQ (mean factor score −0.417) and informational blog (mean factor score −0.423). Titak and Roberson (2013) also found that reader comments were on the personal narrative pole, closer to e-mail and blogs, rather than on the informational pole.

Dimension 2 “Informational-argumentative” is the most characterising dimension for online news comments in this analysis: with a mean factor score of 0.613, they are one of the most representative registers on Dimension 2. They are clearly marked by the co-occurrence of nominalisations, a high average word length, conjuncts, *that* verb complements, and suasive verbs. As already mentioned in the previous section, all of these features contribute to creating informational and argumentative discourse. The other registers which are most representative of Dimension 2 are research articles (mean factor score 1.404) and FAQ (mean factor score 0.485)—both highly information-focused registers with an argumentative structure. The registers closest, and therefore most similar, to online news comments on this dimension are FAQ and informational blog (mean factor score 0.296), both marked by an informational-argumentative style, even though to a lesser extent than online news comments.

In regard to Dimension 3 (we remind the reader that the interpretation of this dimension is not conclusive) online news comments are rather instructional than narrative-descriptive. That said, their mean factor score on Dimension 3 is close to zero, which means that neither the features on the negative pole nor the features on the positive pole of this dimension are highly characteristic of online news comments. Typical instructional registers in this dataset are FAQ, description for sale, advice, and recipe. These registers are marked by a large amount of present tense forms and, to a lesser extent, second person pronouns and possibility modals. Registers representative of the narrative-descriptive pole are description of a person, personal blog, and sports report, which are marked by the co-occurrence of past tense verbs, third person pronouns, and public verbs. The registers most similar to online news comments (mean factor score −0.127) are research articles (mean factor score −0.045) and opinion blog (mean factor score −0.135) on the negative pole of Dimension 3, while the closest registers on the positive pole are religious sermon (mean factor score 0.044) and encyclopedia article (mean factor score 0.201).

According to their location on the three dimensions of variation, online news comments can best be characterised as instances of informational-argumentative discourse with a slight involved-evaluative slant. Anyone with experience reading online news comments will find this description apt: They tend to range from the preachy to the encyclopedic, with heavy argumentation. This characterisation is certainly intuitive if we consider the situational context in which online news comments are produced. Online news comments invite users to communicate their opinion on current news issues and can therefore contain involved and evaluative features (as indicated by their position on Dimension 1). However, online news comments are not subject to on-line production constraints and can be revised before posting, so that information can be integrated and commenters can make precise lexical choices to make their arguments (as indicated by their position on Dimension 2). This description is also in line with our other recent analyses. Ehret and Taboada (2020) compared online news comments to traditional written and spoken registers and found that they are strongly evaluative in nature, combining argumentative, informational, and some involved features (Ehret and Taboada, 2020, 23), while Cavasso and Taboada (2021) observe their overwhelmingly negative nature, with personal affective opinion (*I hate the candidate*) eschewed in favour of more detached evaluation (*The candidate is incompetent; The candidate’s policies are bad*). As illustrated in (4), online news comments can thus range from involved-evaluative to involved-argumentative and informational-argumentative. In our analysis of exclusively written online registers, however, online news comments are not as prominently evaluative as other online registers and their evaluative nature did not emerge as a separate dimension of variation.

(4) a. I_1st person pronoun‘_m_contraction_ very_amplifier_ flattered that my writing is_be main verb_ so_emphatic_ powerful_predicative adjective_ it scares you_2nd person pronoun_. (comments, comments_33450158_18 _MAT.txt).

b. I_1st person pronoun_ agree_public verb_ that_that verb complement_ more controlled peer reviewed research still needs to be done but let’s_contraction_ not run around saying_public verb_ that_that verb complement_ there is 0 scientific evidence. (comments, comments_7018634_53_MAT.txt)

c. However_conjunct_, the SCC quite_adverb_ often_adverb_ throws back legislation_nominalisation_ to the government_nominalisation_ to redraft or abolish. (comments, comments_24630480_7_MAT.txt)

A large body of literature has explored the abusive and toxic nature of much online content and news comments in particular (McGuire, 2015; Gardiner et al., 2016; Muddiman and Stroud, 2017; Wolfgang, 2018; Juarez Miro, 2020). We found some toxicity in the comments in our corpus (Kolhatkar and Taboada, 2017a; Gautam and Taboada, 2019; Kolhatkar et al., 2020), but a relatively small amount, likely because the newspaper uses both automatic and human moderation to filter out the worst abuse.

Our previous analyses compared online news comments to other traditional registers (Ehret and Taboada, 2020), showing that they are not conversational at all. Here, we explore online registers in general and find that the nature of online registers is quite different from traditional written and spoken registers, and that comments are unique in the space of online registers. On the one hand, online registers are substantially more evaluative than traditional written registers—hence, online news comments do not emerge as strongly evaluative in this analysis. Although the fundamental distinction between involved and informational discourse (Biber, 1988) is still present in online registers, the scale of this continuum differs from analyses of purely traditional registers. On the other hand, online registers—and therefore also the emerging dimensions presented in this paper—seem not as clearly delineated as traditional registers in that they tend to combine features customarily associated with several (traditional) registers, and/or written and spoken language (Biber et al., 2015; Egbert et al., 2015). They are involved, like spoken language, but informational and argumentative like many written registers.

Our results contribute to the growing body of evidence that online registers are a different form of communication, and not a hybrid mode somewhere between speech and writing. Studies of Twitter (Clarke and Grieve, 2019), Reddit (Liimatta, 2019), and other online platforms (Hardy and Friginal, 2012; Titak and Roberson, 2013; Pavalanathan et al., 2017; Berber Sardinha, 2018), point to a new type of communication, including individual variation within the various platforms and communication channels. For instance, Liimatta (2019) found the now-familiar informational style in Reddit posts, but also, similar to the present analysis, an instructional focus. Berber Sardinha (2018) discovered two different types of stance (evidentiality and affect) in a study of a mix of online registers. Titak and Roberson (2013) placed reader comments in a personal narrative space (with orientation to the past) and also found that they tend to be involved. Hardy and Friginal (2012) found, like us and most other MDA studies, an informational vs. involved dimension in their analysis of blogs. Unlike the present paper, and due to the personal and narrative style of blogs, they additionally found addressee focus and narrative style dimensions. This makes perfect sense, as each platform and communication medium serves different communicative purposes, has different affordances, and is built around different communities of practice. Thus, the online space can be best described as a “continuous space of register variation” (Biber and Egbert, 2018, 196).

We should point out, before concluding, that our study is firmly language-dependent. The two corpora analysed are in English and it is quite possible that other languages may differ in the dimensions exhibited by different types of online registers. Biber (1995) shows that the main dimensions are constant across languages, especially the first dimension repeatedly found in multi-dimensional analyses (involved vs. informational). That result applies, however, to traditional written and spoken registers and may not be as robust in the online context.

## 5 Conclusions

This paper presented an analysis of online news comments in the context of other online registers. In particular, we conducted an MDA analysis to explore the linguistic features of online news comments compared to an extensive set of common online registers such as personal blog, advice pieces, or reviews.

Describing the position of online news comments along the three emerging dimensions, “Involved-evaluative”, “Informational-argumentative”, and “Narrative-descriptive vs. instructional”, our results corroborate previous research on online news comments. A recent publication established online news comments as a separate register strongly different from other traditional written and spoken registers and described them as argumentative and evaluative instances of discourse (Ehret and Taboada, 2020). Although in the present analysis online news comments also turned out to combine an argumentative-informational style with some involved-evaluative characteristics, we found that online news comments are by far not as involved and evaluative as other online registers.

The analysis presented here thus further refines the previous description of online news comments and allows two general conclusions: First, online registers are not as clearly defined as traditional registers, because they combine features typically found in spoken and informal language with features typical of writing and formal language as well as feature combinations from multiple registers. Second, online registers tend to be more involved and evaluative than traditional registers. Although some online registers have consistently been shown to be involved (e.g. personal blog, advice) vs. other, more informational registers (e.g. research articles, informational blog), it is the involved plus evaluative makeup of online registers which marks them as distinct from other (traditional) registers. This unique combination of evaluative or opinionated features with informational, narrative, and descriptive styles has been previously noted and contributes to the hybrid nature of online registers (Biber and Egbert, 2016; Biber et al., 2015, for hybridisation of online registers see also; Santini, 2007).

These two general characteristics, their unique mix of spoken and written features combined with the involved-evaluative characteristics, suggest online registers are a different mode of communication, neither spoken nor written, and certainly not somewhere in the middle.

## Data Availability

Publicly available datasets were analyzed in this study. This data can be found here: https://github.com/sfu-discourse-lab/SOCC. Code for the study: https://github.com/sfu-discourse-lab/MDA-OnlineRegisters.

## Footnotes

1 ‘Description for sale’ was originally labelled in CORE as ‘Description with intent to sell’. We have shortened the label.

2
https://github.com/sfu-discourse-lab/MDA-OnlineRegisters

3 The tagger is based on and requires the Stanford part-of-speech tagger (Toutanova et al., 2003).

4 This would be considerably low if our primary interest was in variable reduction. However, the focus here is on the interpretability of the factors and the description of online news comments.
