# Supplementary material for: Characterising Online News Comments: A Multi-Dimensional Cruise Through Online Registers
Source: Front Artif Intell. 2021 Jun 14;4:643770. doi: 10.3389/frai.2021.643770 (PMC8238081; doi:10.3389/frai.2021.643770)
Supplement: Supplementary file 1 [file DataSheet1.pdf]

Characterising online news comments: A multi-dimensional cruise through online registers Supplementary material

Katharina Ehret and Maite Taboada

Discourse Processing Lab, Department of Linguistics, Simon Fraser University, Burnaby, BC, Canada

Correspondence: Katharina Ehret, kehret@sfu.ca

Frontiers in Artificial Intelligence, May 2021, Volume 4, Article 643770, https://doi.org/10.3389/frai.2021.643770

1 SUPPLEMENTARY TABLES AND FIGURES

1.1 Tables

Table S1: Part-of-speech tags and corresponding part-of-speech.

POS-tag	Corresponding part-of-speech
AMP	Amplifiers
ANDC	Non-phrasal coordination
AWL	Average word length
BEMA	BE main verb
BYPA	BY passive
CAUS	Causal subordinator cause
CONC	Concessive subordinator
COND	Conditional subordinator
CONJ	Conjuncts
CONT	Contractions
DEMO	Demonstratives
DEMP	Demonstrative pronouns
DPAR	Discourse particles
DWNT	Downtoners
EMPH	Emphatics
EX	Existential THERE
FPP1	1st person pronouns
GER	Gerunds
HDG	Hedges
INPR	Indefinite pronouns
JJ	Attributive adjectives
NEMD	Modals necessity
NN	Nouns
NOMZ	Nominalisations

OSUB	Other subordinator
PASS	Agentless passives
PASTP	Past participle clauses
PEAS	Perfect aspect
PHC	Phrasal coordination
PIN	Prepositions
PIRE	Pied-piping relatives
PIT	Pronoun IT
PLACE	Place adverbials
POMD	Modals possibility
PRED	Predicative adjectives
PRESP	Present participal clauses
PRIV	Private verbs
PRMD	Modals predicitive
PROD	DO as pro-verb
PUBV	Public verbs
RB	Adverbs
SERE	Sentence relatives
SMP	SEEM/APPEAR
SPAU	Split auxiliaries
SPIN	Split infinitives
SPP2	2nd person pronouns
STPR	Stranded prepositions
SUAV	Suasive verbs
SYNE	Synthetic negation
THAC	THAT adjective complements
THATD	THAT deletion
THVC	THAT verb complements
TIME	Time adverbials
TO	Infinitives
TOBJ	THAT relatives obj.
TPP3	3rd person pronouns
TSUB	THAT relatives subj.
TTR	Type-token ratio
VBD	Past tense
VPRT	Present tense
WHCL	WH clauses
WHOBJ	WH relatives obj.
WHQU	WH questions
WHSUB	WH relatives subj.
WZPAST	Past participle WHIZ deletion
WZPRES	Present participle WHIZ deletion
XX0	Analytic negation

Table S2: Promax-rotated model with three factors. Loadings were rounded to the third decimal point. Positive values indicate co-occurrence of the features; negative values indicate complementary distribution.

Feature	Factor 1	Factor 2	Factor 3
AMP	0.179	0.052	0.022
ANDC	0.107	0.005	-0.029
AWL	-1.036	0.652	-0.062
BEMA	0.547	0.024	-0.006
BYPA	-0.242	0.151	0.060
CAUS	0.256	0.106	0.074
CONC	0.139	-0.031	0.040
COND	0.423	-0.002	-0.175
CONJ	-0.157	0.347	-0.073
CONT	0.735	-0.223	-0.057
DEMO	0.185	0.143	-0.068
DEMP	0.330	0.180	-0.052
DPAR	0.226	-0.081	0.002
DWNT	0.089	0.036	-0.012
EMPH	0.449	-0.086	-0.069
EX	0.171	0.086	-0.030
FPP1	0.708	-0.258	0.137
GER	-0.251	0.093	-0.058
HDG	0.281	-0.091	0.005
INPR	0.163	0.051	0.023
JJ	-0.497	0.319	-0.187
NEMD	0.066	0.229	-0.088
NN	-0.737	-0.235	-0.069
NOMZ	-0.706	0.716	-0.093
OSUB	0.027	-0.023	0.026
PASS	-0.272	0.266	0.139
PASTP	-0.189	-0.043	-0.014
PEAS	-0.082	0.078	0.195
PHC	-0.462	0.145	-0.141
PIN	-0.640	0.018	-0.023
PIRE	-0.129	0.155	0.012
PIT	0.484	-0.049	0.013
PLACE	0.048	-0.184	0.002
POMD	0.263	0.062	-0.206
PRED	0.350	0.195	0.005
PRESP	-0.188	-0.014	0.011
PRIV	0.460	0.094	0.107
PRMD	0.252	-0.072	-0.176
PROD	0.398	-0.076	0.006

PUBV	-0.065	0.185	0.321
RB	0.599	-0.004	-0.014
SERE	-0.129	0.056	0.015
SMP	0.105	0.033	0.029
SPAU	0.083	0.118	-0.030
SPIN	0.038	0.080	-0.022
SPP2	0.445	-0.092	-0.260
STPR	0.293	-0.111	0.023
SUAV	-0.044	0.296	0.052
SYNE	0.129	0.132	0.020
THAC	0.075	0.129	0.028
THATD	0.334	0.036	0.264
THVC	-0.031	0.355	0.148
TIME	0.143	-0.193	0.144
TO	0.275	0.092	-0.078
TOBJ	0.019	0.193	0.094
TPP3	0.223	-0.069	0.375
TSUB	-0.129	0.209	-0.054
TTR	0.036	0.214	0.063
VBD	0.057	-0.083	0.983
VPRT	0.555	0.097	-0.523
WHCL	0.259	0.028	0.027
WHOBJ	0.003	0.034	0.016
WHQU	0.152	0.073	-0.058
WHSUB	-0.031	0.120	0.054
WZPAST	-0.379	0.181	0.006
WZPRES	-0.155	-0.005	-0.024
XX0	0.571	0.130	0.030
